# Trends in Between-Country Health Equity in Sub-Saharan Africa from 1990 to 2011: Improvement, Convergence and Reversal

**DOI:** 10.3390/ijerph13060620

**Published:** 2016-06-22

**Authors:** Jiajie Jin, Di Liang, Lu Shi, Jiayan Huang

**Affiliations:** 1 Key Laboratory of Health Technology Assessment, Ministry of Health, Fudan University, Shanghai 200032, China; 15211020032@fudan.edu.cn; 2 Department of Health Policy and Management, University of California, Los Angeles, CA 90095, USA; lesley200712666@sina.com; 3 Department of Public Health Sciences, Clemson University, Clemson, SC 29631, USA; lus@clemson.edu

**Keywords:** health inequity, Africa, convergence trend, under-5 mortality rate, life expectancy

## Abstract

It is not clear whether between-country health inequity in Sub-Saharan Africa has been reduced over time due to economic development and increased foreign investments. We used the World Health Organization’s data about 46 nations in Sub-Saharan Africa to test if under-5 mortality rate (U5MR) and life expectancy (LE) converged or diverged from 1990 to 2011. We explored whether the standard deviation of selected health indicators decreased over time (*i.e.*, sigma convergence), and whether the less developed countries moved toward the average level in the group (*i.e.*, beta convergence). The variation of U5MR between countries became smaller from 1990 to 2001. Yet this sigma convergence trend did not continue after 2002. Life expectancy in Africa from 1990–2011 demonstrated a consistent convergence trend, even after controlling for initial differences of country-level factors. The lack of consistent convergence in U5MR partially resulted from the fact that countries with higher U5MR in 1990 eventually performed better than those countries with lower U5MRs in 1990, constituting a reversal in between-country health inequity. Thus, international aid agencies might consider to reassess the funding priority about which countries to invest in, especially in the field of early childhood health.

## 1. Introduction

During recent decades, the economies of many countries in Sub-Saharan Africa, a region which ranked at the lowest stratum of population health in the world, have improved substantially. In the meantime, domestic governments and various external funders have committed to strengthen local health systems in this region [1]. As average health indicators of the region improved, one question remains: Has health inequity been reduced over time along with economic development and increased investments?

Eliminating health inequity is a major and important goal for public health [2,3]. Thus, a number of previous studies have used various metrics to track health inequities in Sub-Saharan Africa [4,5,6]. Two popular approaches in this area are comparing cross-sectional health indicators between people in the wealthiest quartile and the poorest quartile [7], and comparing health disparities across different time periods [8]. However, previous studies have various limitations. First, most previous studies tended to use indicators based on health financing data (such as health expenditure and public finance data) [9,10] and health service delivery data (such as health service access) [11,12]. In addition, previous studies were mostly limited to a few countries that had sufficient data on national health care systems and population health, such as Ghana, South Africa, and Tanzania [13,14]. In addition, women and children, instead of the general population, were more commonly studied than other groups [15,16]. Thus, most previous studies contributed to identifying individual-level factors associated with health inequity within a country, but were mostly silent about the between-country health inequity in this region.

In order to study the trends in between-country health inequity in Sub-Saharan Africa, we applied the concept of convergence to examine whether health outcome indicators converge or diverge across countries over time. The theory of convergence came from neoclassical growth model [17,18]. It has been extensively used in economics to examine the convergence within countries or regions based on macro-indicators such as gross domestic product (GDP) and income [19]. Since the 1990s this concept has been applied in public health, usually for analyzing the convergence trend in variation of health care expenditures in North America and Europe [20,21]. 

Few studies have used convergence methods to examine variations in health outcome indicators, such as life expectancy and infant mortality in Sub-Saharan Africa. Clark found that during the 1955–2005 period, life expectancy averages converged, but that infant mortality rates continuously diverged across the world [22]. Clark also found that the worldwide divergence of infant mortality might be associated with the fact that Sub-Saharan Africa had the highest infant mortality but had the lowest reduction in infant mortality [22]. Neumayer specifically looked at the impacts of HIV/AIDS on the cross-national convergence in life expectancy, infant mortality, and under-5 mortality in the world [23]. By using both historical and projection data, Neumayer found that the recent lack of cross-national convergence in life expectancy is largely attributable to HIV/AIDS in Sub-Saharan Africa [23]. But the epidemic has not stalled convergence in infant and child survival rates, though it has slowed convergence. However, Clark and Neumayer did not look at the convergence within Sub-Saharan Africa. Thus, our study is among the early attempts that track between-country health inequity in Sub-Saharan Africa using the convergence methodology.

We hypothesized that the between-country health inequity among African countries would decrease over time, given that foreign aid in health care tended to go to those countries with lowest level of health outcomes. The alternative hypothesis was that the between-country health inequity among African countries would increase over time. The rationale was that although both macroeconomic development and foreign investments for health would have presumably desirable impact on the population health in Sub-Saharan Africa, their effects on removing health disparity might not be monotonous or linear. Rapid industrialization could improve the health care services among those originally richer while worsen the environment for those already poorer, and foreign aid in certain geographic spots could attract local resources away from those neediest regions.

## 2. Materials and Methods 

### 2.1. Metrics Used for Tracking Health Inequity

Sigma and beta convergence are the two popular metrics used for measuring inequity. Sigma (σ) convergence occurs when the standard deviation of the study variable decreases over time. Beta (β) convergence occurs when the less developed entities grow more quickly than their developed counterparts, moving toward the average level of development in the group. Beta convergence is also known as “regression to mean”. In line with the convergence concept, the existence of beta convergence is a necessary condition for the existence of sigma convergence [18]. In health terms, existence of beta convergence means the initially below-average countries improve their health status faster than the above-average countries. As those initially below-average countries move closer toward the mean, the sigma convergence then emerges. Thus, the health gap between countries would become narrower and health inequity would decrease over time.

In this article, variation between countries in under-5 mortality rate (U5MR) and life expectancy (LE) were selected as measure to describe between-country health inequity in Sub-Saharan Africa. Reducing U5MR is one of the Millennium Development Goals (MDG) [24]. U5MR was widely used to measure the survival of children, and it could account for 90% of deaths of children under 18 years of age [4,25]. U5MR was sensitive to structural changes and rising epidemics that affect the general population. This indicator could sometimes be used to identify who are the vulnerable groups when researchers lack access to data of disease incidence and prevalence. Life expectancy could reflect the social, economic and environmental conditions in which people live in, including health care services. From the viewpoint of research feasibility, we used these two indicators in our analysis partly because they are official statistics available for all countries in the region for all years, while we are aware of the risk that some of these country-level statistics might not be very accurate on a year-to-year basis.

### 2.2. Analysis Model

In this study, we looked at both sigma convergence and beta convergence metrics to track the variation in selected health indicators (U5MR and LE). For sigma convergence, in order to eliminate the influence of the measurement scale and dimension of health indicators, we chose the coefficient of variation as the representative of standard deviation:

(1) $$CV_{t}=\left(\frac{SD_{t}}{MN_{t}}\right)\times 100\%$$

where $CV_{t}$ is the value of coefficient variation for n countries at time t, SD is the standard deviation, and MN is the mean. The falling of CV value for certain health outcome measure during the sample period (CVt + 1 < CVt) indicates the presence of sigma convergence.

For beta convergence, we used the following model:

(2) $$Y=\alpha +\beta X_{i,t}+\gamma C_{i,t}+\varepsilon_{i,\;t}+u_{i,t}$$

The econometric model we used here for beta convergence exploration is a random effects model where $\varepsilon_{i,t}$ stands for within-country error and $u_{i,t}$ stands for between-country error as presented in Equation (2), where X represents the natural logarithm of certain health indicator in country *i* at time *t*, and Y is given by $Y=\frac{1}{T}\ln\left(\frac{y_{i,t+T}}{y_{i,t}}\right)$, the growth rate per year from time *t* through time *t* *+* *T*. 

A negative and significant value for β means the presence of beta convergence. In the formula $\beta =\frac{1-e^{-bT}}{T}$, the value of b implies the convergence rate. 

$C_{i,t}$ is a vector including the matrix of five control variables of country *i* at time *t*. When the vector $C_{i,t}$ is empty, this formula calculates absolute beta convergence. Otherwise, the conditional beta convergence is calculated. Our control variables include: (1) the natural logarithm of gross domestic product per capita of country *i* at time *t* ($GDP\_PC_{i,t}$), to control for different economic development level of countries; (2) the natural logarithm of health care expenditure per capita of country i at time t ($HE\_PC_{i,t}$), to control for domestic investments in health; (3) the natural logarithm of development assistance for health per capita of country i at time t ($DAH\_PC_{i,t}$), to control for external health investments from international communities; (4) urban population as share of total population of country *i* at time *t* ($UrbanPop_{i,t}$), which is known to influence health service provision [26]; (5) HIV prevalence rate ($HIV-PR_{i,t}$).

In our analysis, we performed long-run single regression based on the cross-sectional data collected at the initial year and the terminal year. Panel estimate was also used by each 5-year period, 1990–1994, 1995–1999, 2000–2005, and 2006–2011, to allow for period effects.

### 2.3. Data Source

The World Health Organization’s Africa region consists of 47 nations. Since the Republic of South Sudan declared its independence as late as July 2011, it was excluded from the data analysis. We collected the time-series data of 46 countries to form a sample of 1012 observations (46 countries × 22 annual observations). Data of GDP and HE were adjusted to 2011 U.S. dollar by purchase power parity. The U5MR, LE, GDP-PC, and HE-PC data for the 46 Africa countries were collected from the Union Nation Database (UNdata). 

DAH data were extracted from “Financing Global Health 2013”, published by Institute for Health Metrics and Evaluation, University of Washington. DAH in each year equals the sum of gross yearly disbursements on all health-sector grants and loans, and health related program expenditures. DAH aims to achieve either country-specific health improvements or to finance health-related global public goods [27]. 

Data of HE-PC from 1990 to 1994 were not available. Thus, we used the data of HE-PC in 1995 as proxies for HE-PC from 1990 to 1994, since between 1990 and 1995 there had been no significant economic growth in Sub-Saharan Africa. This is nevertheless a limitation of this analysis as countries like Rwanda could experience significant changes in HE-PC before and after their 1994 genocide.

## 3. Results

### 3.1. Overview of Health Expenditure in Africa

**Figure 1:**
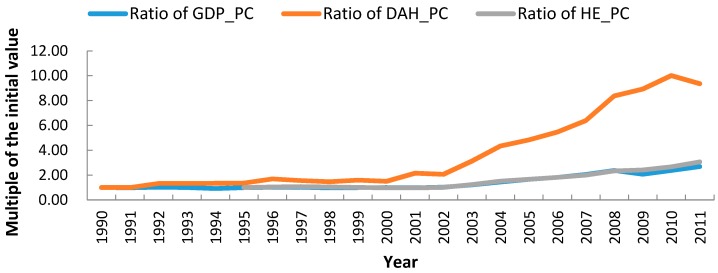
Analysis of trends for GDP per capita, HE per capita and DAH per capita in Sub-Saharan Africa, 1990 to 2011. (1) The ratio of GDP per capita (GDP_PC), DAH per capita (DAH_PC) and health expenditure per capita (HE_PC) for each year is equal to the value of the corresponding variable in each year divided by the initial value; (2) For ratio of GDP per capita and ratio of DAH per capita, the value of the corresponding variable in 1990 is used as the initial value; (3) As to HE-PC, the value of the corresponding variable in 1995 is used as the initial value.

In 2011, the average GDP per capita in Sub-Saharan Africa was 3076 United States dollars, substantially up from the average value of 1145 U.S. dollars in 1990 (adjusted to 2011 U.S. dollars by purchase power parity). DAH per capita in Africa grew from 1.00 to 9.37 U.S. dollars during that period, representing a much faster increase than the per capita GDP. Figure 1 showed the growth curves of these indicators as compared with initial values. The growth of these indicators notably accelerated after 2000. Moreover, DAH per capita grew faster than that of GDP per capita and heath expenditure per capita, while the latter two indictors had a similar rate of growth. This outcome implied that external health aid outpaced domestic health investment, especially after 2000.

### 3.2. Variation of Health Outcomes in Africa

**Figure 2:**
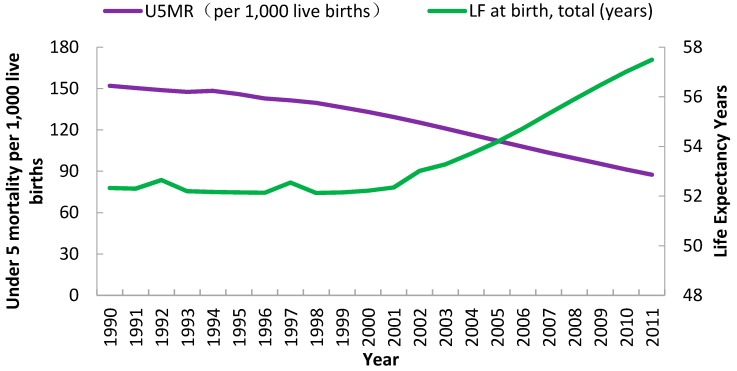
Analysis of trends for U5MR and LE in Sub-Saharan Africa, 1990 to 2011. U5MR relates to purple graphing line and corresponds to the left-hand Y-axis, while LE relates to green graphing line and corresponds to the right-hand Y-axis.

U5MR in Sub-Saharan Africa trended downward from 151.97/1000 live births for 1990 to 87.54/1000 live births for 2011, representing about a decrease of 40% from the initial value (Figure 2). The first 10% decrease occurred from 1990 to 1999, and the decrease accelerated after 2000. Life expectancy at birth grew from 52.73 years in 1990 to 57.49 years in 2011. The absolute value of this indicator had no consistent increase until 2000. After that, LE in Africa showed a consistent upward trend (Figure 2).

**Figure 3:**
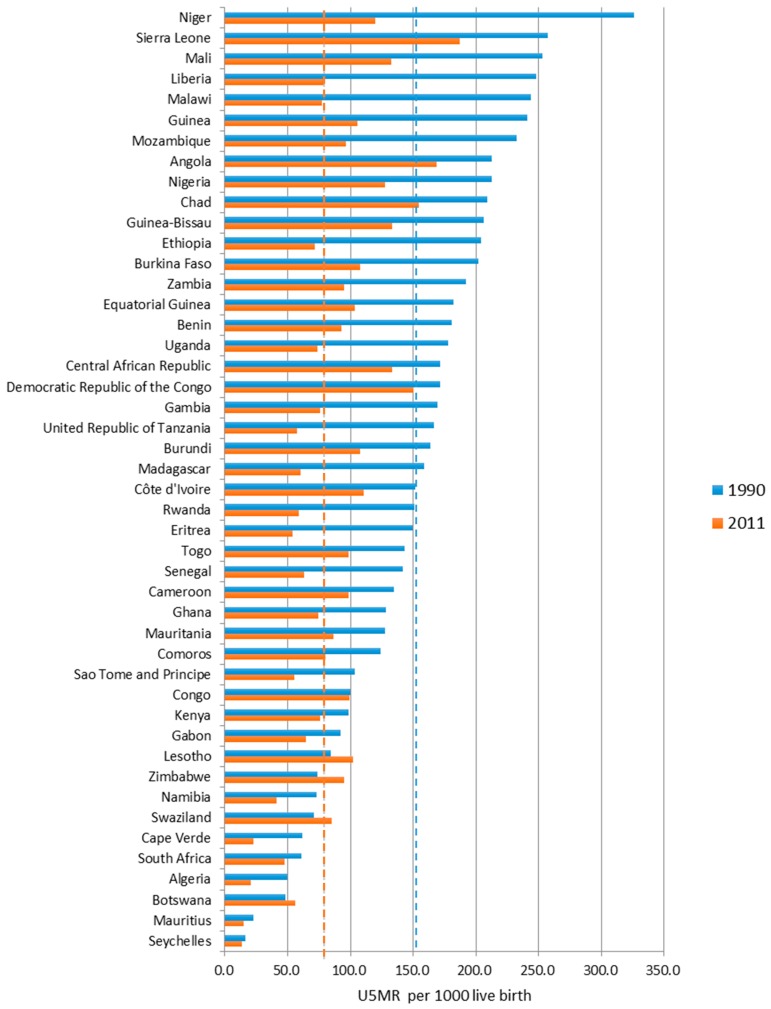
The variation for U5MR of African countries, between 1990 and 2011. (1) In figure, the red dotted line is the average value for 2011, 87.54 per 1000 liver birth, while the blue dotted line represent the average value for 1990, 151.97 per 1000 liver birth; (2) The countries were sorted by U5MR for 1990.

Figure 3 shows the U5MR of each Sub-Saharan African country in 1900 and 2011. Although the Sub-Saharan Africa region as a whole witnessed a consistently downward trend in U5MR (Figure 2), the trends for individual countries were somewhat different. Nine countries had a sharp downward trend for U5MR—more than 60% decrease—especially in countries like Malawi, Liberia, Tanzania, Ethiopia, Eritrea, and Niger. Although these countries initially had a very high U5MR, the 2011 values for these countries were about the same as or even less than the average value for the region. We noticed that not only has the overall level of U5MR improved, the range between the country with the highest U5MR and the one with the lowest has also narrowed. In 1990 the range was 309.5, with the highest value of 326.1 per 1000 live birth (Niger) and the lowest value of 16.6 (Seychelles). The range narrowed to 173.9 in 2011, with the highest value of 187.2 per 1000 live birth (Sierra Leone) and the lowest value of 13.3 in Seychelles. 

**Figure 4:**
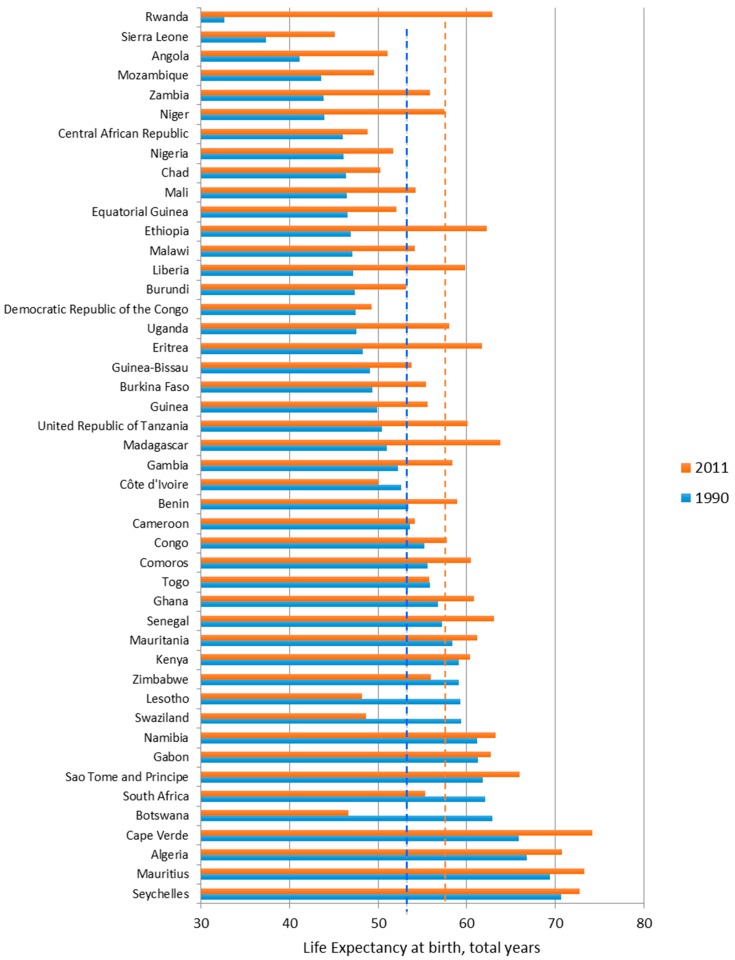
Life Expectancy of African countries, 1990 and 2011. (1) In figure, the red dotted line is the average value for 2011, 57.49 year old, while the blue dotted line represent the average value for 1990, 52.73; (2) The countries were sorted by LE for 1990.

For the outcome of life expectancy, many countries that had lower LE in 1990 appeared to have improved faster than those with higher initial LE (Figure 4). In the case of Rwanda, Ethiopia, Niger, Eritrea, Liberia, LE of these countries in 1990 were much lower than the average value for the entire region, yet LE of these countries in 2011 was higher than the average value.

**Figure 5:**
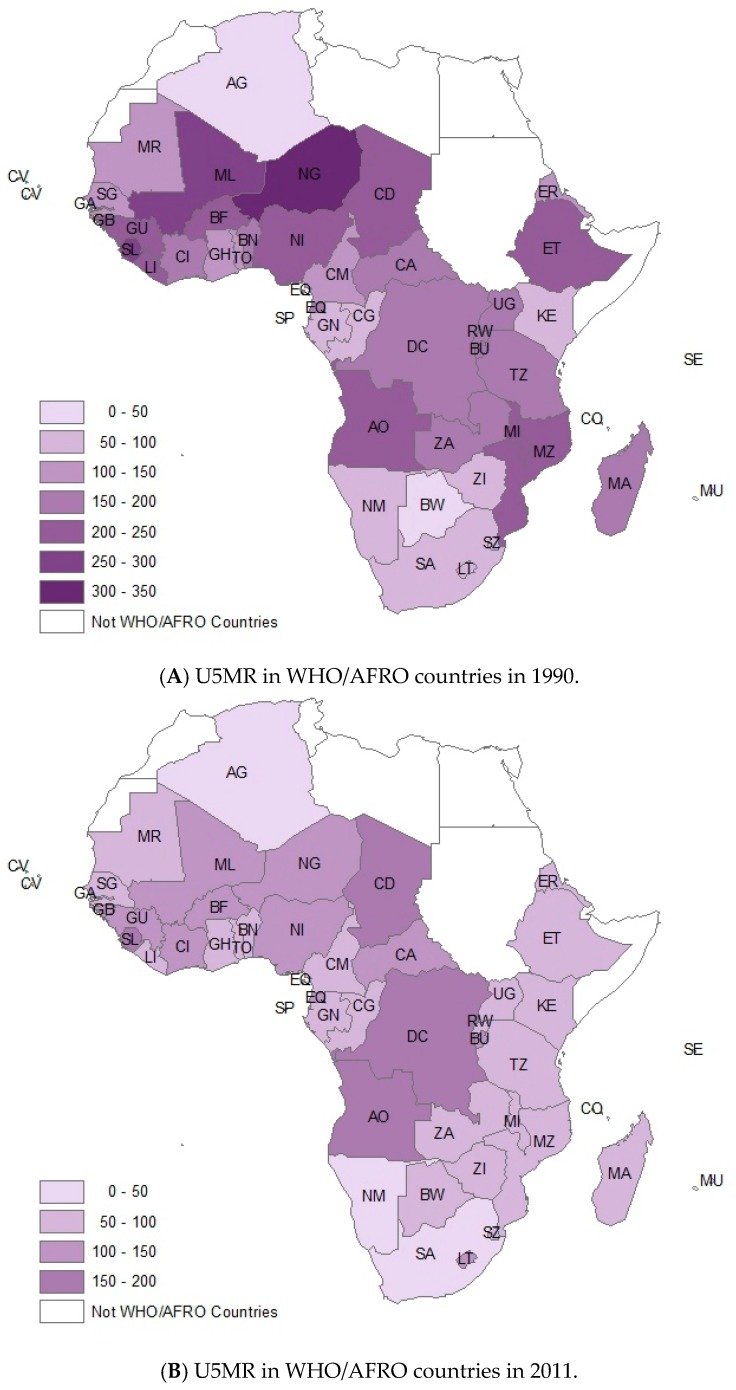
Comparing the variation of U5MR of Africa Countries, (**A**) 1990 and (**B**) 2011, by GIS. This figure showed the variation of U5MR by GIS.

Mapping based upon Geographic Information System (GIS) was also used to demonstrate the different variation between areas. Figure 5 showed the variation of U5MR. In the entire region, there were only 4 countries which showed the upward trend of U5MR from 1990 to 2011, including Zimbabwe (ZI, with 28.65% upward), Lesotho (LT, 20.83% upward), Swaziland (SZ, 19.75%), and Botswana (BW, 16.67%). More important, all of these four countries were in the southern Africa. On the other hand, U5MR of the other 42 countries demonstrated downward trends, albeit with different paces of decrease. The countries with highest downward rate of U5MR were mostly located in the east of Africa, including Malawi (MI, with 68.35% decrease), Tanzania (TZ, with 65.35% decrease), Ethiopia (ET, 64.75% decrease), Eritrea (ER, 64.09%), Madagascar (MA, 61.77%), and Rwanda (RW, 60.97%).

**Figure 6:**
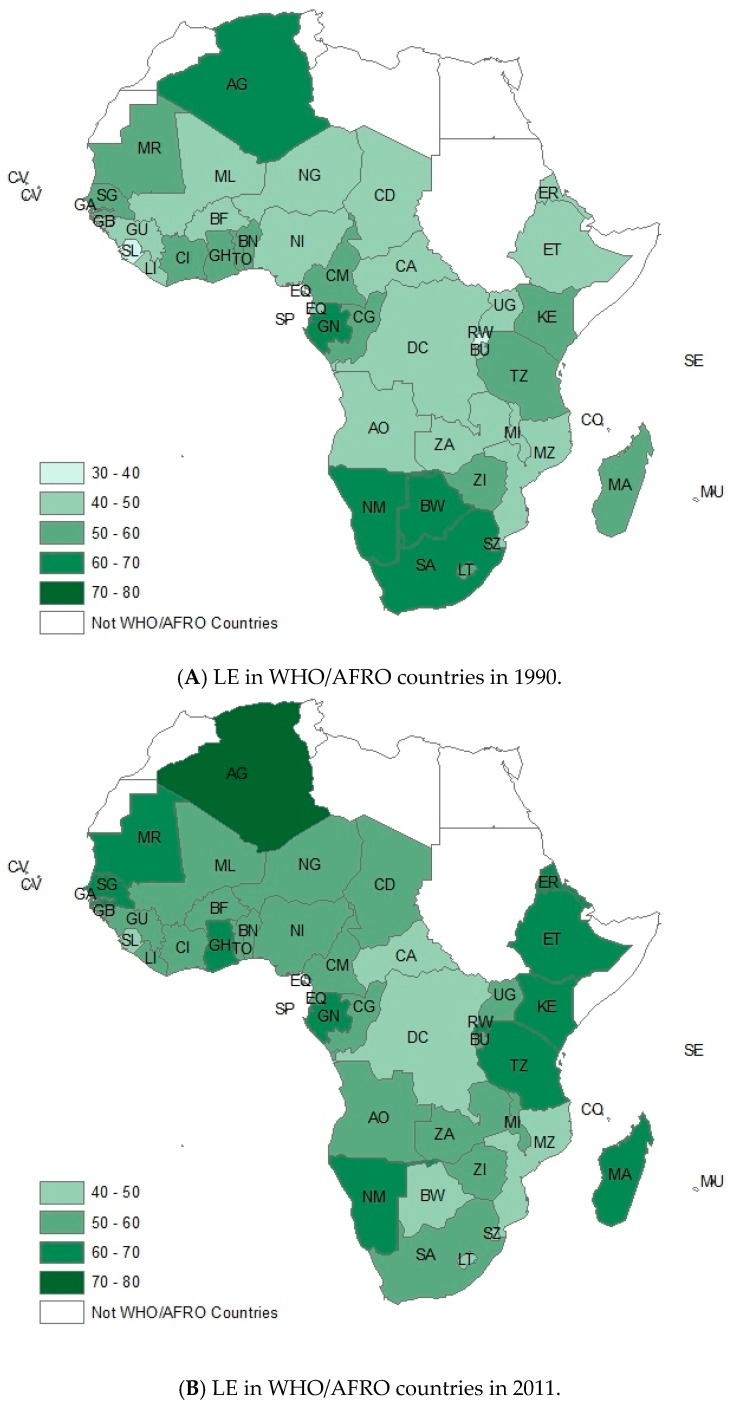
Comparing the variation of LE of Africa Countries, (**A**) 1990 and (**B**) 2011, by GIS. This figure showed the variation of LE by GIS.

For the outcome of life expectancy, there were six countries which showed a downward trend of LE, and five of them are located in southern Africa (Figure 6), including Botswana (BW, 25.85% decrease), Lesotho (LT, 18.73% decrease), Swaziland (SZ, 18.00% decrease), South Africa (10.99% decrease), and Zimbabwe (ZI, 5.39% decrease), while the countries with steepest upward slopes were mainly located in East Africa, including Rwanda (RW, with 92.95% increase), Ethiopia (ET, 32.61% increase), Eritrea (ER, 27.94% increase), and Madagascar (MA, 25.08%).

### 3.3. Convergence Analysis of U5MR 

**Figure 7:**
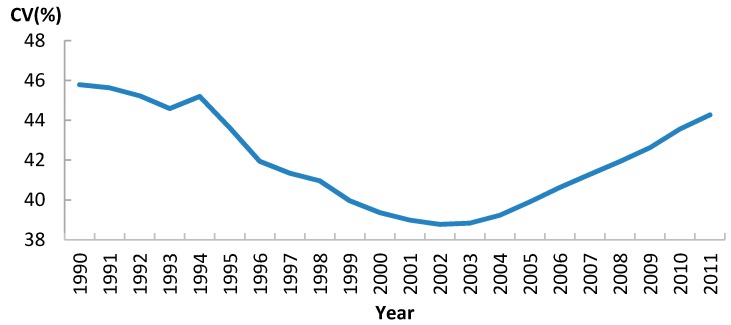
The trend of Coefficient of Variation for U5MR in Africa, 1990–2011.

Although U5MR was declining steadily in Sub-Saharan Africa as a whole region, variations between the 46 Africa countries, in terms of CV, showed a “U” shape curve (Figure 7). This meant that for the first decade of the study period, variations between countries became smaller, indicating a sigma convergence. However, after 2002, the trend moved in the opposite direction, suggesting that variations between countries were getting greater. In other words, there was no continuous sigma convergence of U5MR in Africa from 1990 to 2011 (Figure 7).

As for the beta convergence for U5MR, the coefficient β was equal to −0.050 in absolute beta convergence model with statistical significance (Table 1). This U5MR of 46 countries converged to a steady state, with a weak annual convergent rate (b) around 1%. The coefficient β was insignificant in the conditional model by both simple regression and panel estimates (Table 1), meaning that there was no conditional beta convergence for U5MR when initial disparities between these countries were accounted for.

### 3.4. Convergence Analysis of LE

**Figure 8:**
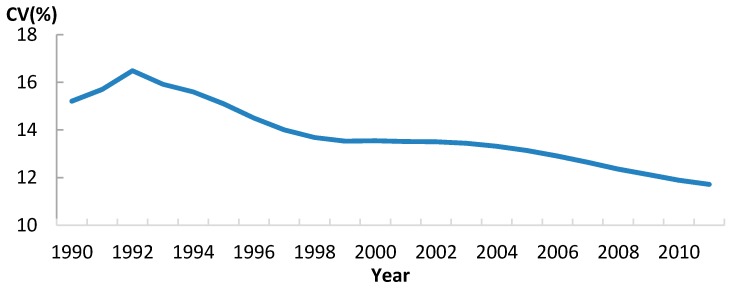
The trend of Coefficient of Variation for LE in Africa, 1990–2011.

For life expectancy in Africa, there was a sharp decreasing trend of CV during the first decade, and this decrease trend of CV continued while the value of LE has notably increased during the second decade (Figure 8). Thus, LE in Sub-Saharan Africa appeared to have a clear sigma convergence, with most values moving toward the increasing mean value.

Based on the outcome of beta convergence, the coefficient B in the absolute model for LE equaled −0.129, with a *p* value smaller than 0.001 (Table 2). It showed the absolute beta convergence trend for LE in Africa from 1990–2011, with the annual convergence rate (b) of 2.24%. Moreover, coefficient β in conditional model was −0.126, also with robust statistical significance. That meant that LE in Africa from 1990–2011 demonstrated the conditional beta convergence trend after controlling for the initial difference between countries.

The result of panel estimates also yielded a statistically significant conditional beta convergence after controlling for the period effect. And the coefficient β is equal to −0.033, with statistical significance (Table 2).

## 4. Discussion

In summary, as for U5MR, there has been a slow absolute beta convergence in Sub-Saharan African region, yet there were no conditional beta convergence or sustainable sigma convergence trends; with regard to LE, we found consistent absolute beta convergence, conditional beta convergence, and sigma convergence. Our findings about LE were similar to the published outcomes by Edwards though he used Theil Index and standard deviation to calculate inequality in LE in Africa [28]. 

One possible explanation for the lack of consistent convergence in U5MR was that a reverse beta convergence emerged later for U5MR in the region. The countries with high initial U5MRs decreased faster than those with low rates, hence the beta convergence emerged. This trend continued even after those countries with high initial U5MRs caught up with those with low initial U5MRs. Thus, countries with high initial U5MRs ended up with a lower U5MR than those countries with low initial U5MRs, constituting a reversal beta convergence. This new disparity between these two groups of countries was not significantly different from the initial disparity in terms of magnitude, except that these two groups of countries swopped their order before and after. This explains the absence of a significant sigma convergence. 

Data availability is a major limitation for this study. Several possible diverging or converging factors for U5MR and LE were considered when we designed this study, such as human resource allocation, health service delivery, disease epidemic, *etc.* But these variables were not readily available for many African countries on an annual basis. For a country-level analysis with relatively small sample size, methods for missing data such as multiple imputation or last-observation-carried-forward are often inappropriate. Thus only economic indicators, including GDP per capita, HE per capita, DAH per capita, and the proportion of urban population, were included in the model as control variables. Thus, it is very important to develop a demographic surveillance system in addressing health inequities [29], and future studies with more enriched data sources could provide us with a fuller picture of what has happened for the between-country health inequity in global health.

It remains debatable whether we need to leave out countries deeply affected by war and genocide such as Democratic Republic of Congo as statistical outliers, as analyses of Sub-Saharan development sometimes leave South Africa out of their analytical framework. We chose to keep Congo in our analysis partly because of the country’s significant weight in Sub-Saharan African population, and plan to include control variables such as war and genocide in our future analyses. 

We started off with the question whether between-country health inequity in Sub-Saharan Africa has been reduced over time, and we made this hypothesis because we expected population health impact from increased foreign investments and economic development. We attempted to explore the impacts of increased foreign investments and economic development on health inequity. However, these relationships can be subtle and should be interpreted with caution. 

Our panel estimates for conditional beta convergence suggested that DAH might be influential in this process. The relationship between DAH and the lack of convergence in U5MR can be complex. As we mentioned before, health investments in Sub-Saharan Africa, especially DAH, have been increasing rapidly after 2000, and countries in this region have also seen an accelerated decline of U5MR since then. It is notable that DAH might have both positive and negative impacts on child survival in this region. Most DAH has been invested on communicable diseases such as HIV, malaria, and tuberculosis, and communicable diseases are one of two main causes for child mortality in this region. Thus, DAH might have contributed to the improved child survival, by alleviating infectious disease epidemics and improving vaccination programs. However, Grepin found negative impacts of health aid on maternal and child health services, since it is not impossible for those newly funded HIV/AIDS treatment programs to divert human resources away from non-HIV maternal and child health care [24]. Furthermore, countries with poor initial health outcomes might be more likely than others to receive external health care aid, and thus have the resources to improve their health conditions, sometimes even at the expense of depleting neighboring countries’ human resources in health care and thus creating a vacuum of health care in their neighbors. Our study does not have sufficient data to test whether there exists this mechanism of improving population health at the expense of neighboring countries, yet it is nevertheless a possible mechanism that development programs need to be wary of. Unfortunately, we cannot further explain the potential relationship between DAH and the reversal beta convergence due to data limitations. 

The reversal in beta convergence discussed above mirrors the trend about HIV prevalence rate in Sub-Saharan Africa: those with initially higher HIV prevalence in 1990 experienced much slower increase in prevalence than those richer countries in southwestern Africa [30]. One encouraging sign for HIV/AIDS control, however, is that those countries with substantial DAH tend to be those who enjoyed accelerated improvement, a preliminary and macro-level signal that supports the effectiveness of DAH programs such as the Global Fund to Fight AIDS, Tuberculosis and Malaria [31] and President’s Emergency Plan for AIDS relief [32]. Those countries that now fell behind the average thus need more evidence-based investment in HIV/AIDS prevention and control, with resources both from outside and from within the Sub-Saharan region [33].

In the meantime, as our data about GDP per capita show, Sub-Saharan Africa has seen impressive economic growth in the 21st century, and the LE has increased concurrently. This may suggest the possible positive impacts of economic development on LE. Economic development’s impact on life expectancy gain is more consistent than its reduction of the child mortality [22], as the latter is more of an indicator of economic disparity rather than the absolute level of economic development. 

However, the region’s success in economic development so far, should not make us infer that effective investments in population health are necessarily following economic progress. Indeed, those countries experiencing life expectancy decrease and stagnating in their U5MR reduction tend to be located in the richer southwestern part of Africa, whereas several economically poorer countries in East Africa rose from low initial ranking and overtook the initially better-off countries in terms of LE and U5MR indicators. Angola’s low standing in U5MR despite its relatively high GDP per capita in the region, for example, means that economic progress does not automatically improve population health. Similarly, Botswana’s economic success and its decline in life expectancy are both extreme examples in African development, hinting that life expectancy does not always follow laudable economic success. All these echo Deaton’s 2010 claim that Gross Domestic Product is a poor measure of welfare to begin with, and thus rapid growth as measured by GDP (as seen in southwestern Africa) should not make people assume that the physical wellbeing necessarily follows those impressive economic growth rate statistics.

## 5. Conclusions

To date, Sub-Saharan Africa still has the lowest overall health indicators among all major regions in the world [34]. It is encouraging to witness that the growth of life expectancy in Sub-Saharan Africa has accelerated after the beginning of the 21st century with a sustained convergence trend, while the U5MR in Africa demonstrates a downward trend. However, the reversal in U5MR convergence signals the need to reconsider priority countries when it comes to international aid, since many initially unhealthiest countries have overtaken some of those initially healthier countries. Of particular concern is that in U5MR many of those currently least healthy countries tend to have larger populations (Democratic Republic of Congo, Angola, Chad, Mali, *etc.*), representing a very substantial proportion of Sub-Saharan African population. For international aid agencies, more priority should be given to those countries that moved from below the average in U5MR backward to above the average during 1990 to 2011 (Botswana, Swaziland, Zimbabwe, Lesotho, Congo, *etc.*). Continuing to exclusively focus on those countries that already achieved a catching-up improvement in U5MR (Malawi, Ethiopia, Tanzania, Rwanda, *etc.*) might miss some more urgent missions elsewhere in the region.

## Figures and Tables

**Table 1:** Beta convergence analysis for U5MR, 1990–2011. **^1,2,3,4^**

Coefficient	Long-Run Single Regression				Panel Estimates	
	Absolute β-Convergence		Conditional β-Convergence		Conditional β-Convergence	
	Value	95% CI	Value	95% CI	Value	95% CI
α	0.138 (0.081)	(−0.025, 0.302)	0.183 (0.292)	(−0.410, 0.775)	−0.036 (0.046)	(−0.127, 0.055)
β	−0.050 * (0.004)	(−0.083, −0.017)	−0.035 (0.030)	(−0.096, 0.026)	0.007 (0.005)	(−0.003, 0.017)
γ						
γ(DAH_PC)			0.003 (0.008)	(−0.014, 0.019)	−0.005 *** (0.001)	(−0.001, 0.000)
γ(GDP_PC)			−0.022 (0.028)	(−0.079, 0.035)	0.002 (0.006)	(−0.008, −0.003)
γ(HE_PC)			0.036 (0.022)	(−0.008, 0.080)	0.001 (0.006)	(−0.009, 0.013)
γ(UrbanPop)			−0.016 (0.024)	(−0.033, 0.065)	0.001 (0.005)	(−0.009, 0.011)
γ(HIV−PR)			−0.014 (0.006)	(−0.001, 0.026)	0.004 ** (0.001)	(0.001, 0.007)
Annual convergence rate (b, %)	3.37					
R^2^	0.174		0.320		0.222	

**^1^** * *p* < 0.05, ** *p* < 0.01, *** *p* < 0.001; **^2^** Standard error is showed in parentheses; **^3^** The country dummies are not reported in the table; **^4^** $\beta =\frac{1-e^{-bT}}{T}$ and time range variable T = 21.

**Table 2:** Beta convergence analysis for LE, 1990–2011. **^1,2,3,4^**

Coefficient	Long-Run Single Regression				Panel Estimates	
	Absolute β-Convergence		Conditional β-Convergence		Conditional β-Convergence	
	Value	95% CI	Value	95% CI	Value	95% CI
α	0.528 (0.078)	(−0.371, 0.685)	0.422 ** (0.100)	(0.219, 0.625)	0.102 *** (0.026)	(0.049, 0.154)
β	−0.129 ** (0.020)	(−0.169, −0.089)	−0.126 *** (0.027)	(−0.180, 0.071)	−0.033 *** (0.007)	(−0.047, −0.019)
γ						
γ(DAH_PC)			0.002 (0.002)	(−0.007, 0.003)	0.001 *** (0.001)	(0.000, 0.002)
γ(GDP_PC)			0.017 * (0.007)	(−0.001, 0.034)	0.004 (0.002)	(−0.001, 0.008)
γ(HE_PC)			−0.017 * (0.005)	(−0.031, 0.004)	−0.003 (0.002)	(−0.008, 0.002)
γ(UrbanPop)			−0.010 (0.007)	(−0.025, 0.004)	−0.002 (0.002)	(−0.006, 0.002)
γ(HIV−PR)			−0.004 * (0.002)	(−0.008, 0.000)	−0.003 (0.001)	(−0.004, −0.001)
Annual convergence rate (b, %)	6.113		6.035		3.054	
R^2^	0.494		0.640		0.307	

**^1^** * *p* < 0.05, ** *p* < 0.01, *** *p* < 0.001; **^2^** Standard error is showed in parentheses; **^3^** The country dummies are not reported in the table; **^4^** $\beta =\frac{1-e^{-bT}}{T}$ and time range variable T = 21.

## Abbreviations

The following abbreviations are used in this manuscript:

DAH_PC	development assistant for health per capita
GDP-PC	Gross domestic product per capita
HE-PC	health expenditure per capita
HIV-PR	HIV prevalence rate
LE	Life expectancy
MDG	Millennium Development Goals
U5MR	under-5 mortality
